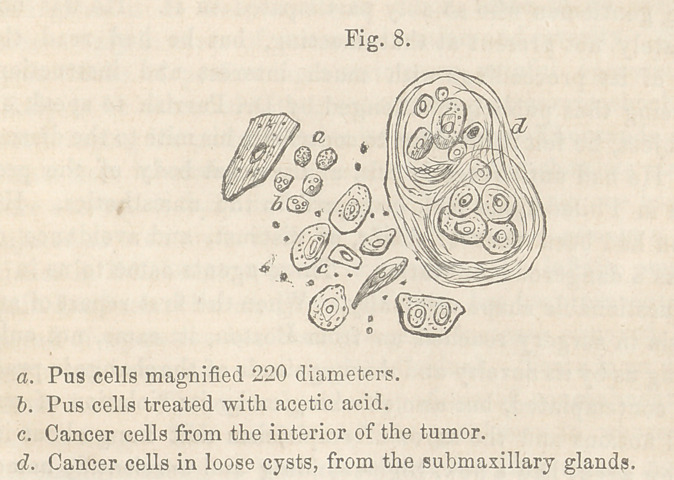# Report of Cases, in Continuation of the Remarks on Epithelial Tumors and Cancer of the Skin

**Published:** 1852-05

**Authors:** J. Da Costa

**Affiliations:** Philadelphia


*Report of Cases, in continuation of the Remarks on Epithelial Tumors and Cancer of the Skin.** By J. Da Costa, M. D., of Philadelphia.

Case I. J. H——, æt. 24, a strong, healthy man, presented himself at the clinic of the Jefferson Medical College, Oct. 5th, 1850, with a tumor on the lower lip, which had been growing for three years. He stated that it commenced as a wart, and that its subsequent increase had never occasioned much pain. Dr. Pancoast removed the whole diseased mass by a V shaped incision; the wound healed kindly, and the patient has remained well ever since.

The tumor, when removed, presented a rough appearance; on its exterior were several red crusts, the interior was hard and nodulated; the mucous membrane lining its internal surface was thickened.

*Microscopical Examination.*—On submitting the crusts on the exterior to the microscope, they were found to consist of dried up epithelial cells, and blood globules. The cells were dark and non-nucleated; (Fig. 3 *a*), acetic acid did not change them.

* The nine cases here selected, were those that seemed to me the most interesting of the 34 cases I spoke of in my previous paper. For the greater part of the specimens, as well as for some particulars about the history of these cases, I am indebted to Drs. Mütter and Pancoast.

Fig. 3

*a.* Old epithelial cells from the surface of the tumor, magnified 200 diam.

*b.* Cells from the interior of the tumor.

*c.* Globular cells.

*d.* Compound granular cells.

A section from the interior of the tumor, made with a Valentin's knife, showed epithelial cells, fibrous cysts, and globular cells. The epithelial cells here were in masses, and not very large; (Fig 3 *b*) their nuclei, however, were distinct; some had nucleoli. The fibrous cysts were, like those represented in Fig. 2, (in the preceding number of the Medical Examiner,) very irregular, and delicate; some were empty, some enclosed epithelial cells. The globular cells, (Fig. 3 *c*) were about 1-4200th part of an English inch in diameter, and mostly non-nucleated. In some of the softer parts of the tumor were seen masses of granules, and very large granular cells, (*d*) with a distinct cell wall.

Case II. Mr. B——, a seafaring man, æt. 43, who had always enjoyed very good health, consulted Dr. Pancoast, February 3d, 1851, for a tumor of the lower lip, that had been coming gradually for two years. He had been much addicted to smoking short pipes. The tumor was removed, February 6th, by the usual incision, and the parts brought together by the twisted suture. The wound healed well, but as the edges subsequently thickened, Dr. Pancoast removed the cicatrix as a matter of precaution. The patient, when last seen, was perfectly cured.

The tumor was small, but, as in the preceding case, rough on the surface and very hard; it had not begun to ulcerate, nor had any red crusts formed as yet on its exterior.

*Microscopical Examination.*—The tumor, examined under the microscope, consisted mainly of epithelial cells, globular cells, and fibrous tissue. The epithelial and globular cells were like those represented (Fig. 3, *b* and *c*); they presented nothing peculiar. The fibrous tissue, however, which existed in great abundance and formed the larger part of the tumor, was remarkably strong: In many places it united to form cysts, most of which were oval. These cysts were formed of very delicate fibres, and contained mostly globular cells. (Fig. 4, *a.*) Some by the interlacing of the fibres, formed in their interior several smaller cysts; (Fig. 4, *b*); in some again the cysts had opened (*c*) and discharged their contents.

Fig. 4.

*a.* Cyst from the interior of the tumor filled with globular cells, magnified 220 diam.

*b.* Oval cyst enclosing three smaller ones.

*c.* Cyst with ruptured wall, and contents discharged.

Case III. H. S——, æt. 52, a robust healthy looking man, had his lower lip parched by the heat of the sun, whilst wheeling wood on the wharf last August. The lip soon became swollen, and a small tumor formed, which gradually enlarged. It did not give rise to much pain, except during sudden changes of temperature. He applied, December 13th, 1851, to the clinic of the Jefferson Medical College, when the whole morbid substance was removed by Dr. Mütter, by a quadrangular incision, and a plastic operation performed to fill up the gap. Previous to the operation there had been some tumefaction of the cervical glands, but this speedily subsided. The wound had united throughout by the 17th of December. March 25th, I saw the patient, and he was in perfect health.

The tumor was about the size of a cherry, and had begun to ulcerate; its exterior was tuberculated; towards the right side was a dark red crust; towards the left, separate from the mass of the tumor, a little hard spot; on the internal surface of the lip there was a circumscribed rough projection.

*Microscopical Examination.*—The crust was composed of dark, long epithelial cells, resembling those in Fig. 3, and mostly without nuclei. Its red color was evidently owing to blood globules, as no blood vessels were perceptible. In a section below this crust, I found epithelial cells, so closely arranged as to resemble fibrous tissue, (see Fig. 2, in preceding number of Examiner), and only discernible by the addition of acetic acid. On placing a portion from the middle of the tumor under the microscope, I found large, distinctly nucleated epithelial cells, free nuclei, and globular cells. In another part, there were a great many free nuclei and granules. The cells in all parts of the tumor, were held together by a very delicate fibrous arrangement, which formed but few cysts. The small, dark, tuberculated spots consisted entirely of granules and globular cells; the separated hard mass contained epithelial cells, fibrous tissue, and granules. The rough circumscribed eminence on the inside of the lip, presented enlarged papillæ, also a few epithelial cells and granular matter.

Case IV. E. J.——, æt. 80, came to the clinic of the Jefferson Medical College, November 12th, 1851, with a large tumor on the lower lip, which had been forming for two years. It gave him no pain, but occasioned great inconvenience in swallowing, especially during the last two months of its growth. He had been in the habit of constantly smoking short pipes. The tumor was removed by Dr. Mütter November 15th. December 1st, the wound had united throughout by the first intention. He left the city December 10th, when last heard of, the disease had not returned.

The tumor, when removed presented very much the appearance of a ripe mulberry; it was about an inch in thickness and occupied the whole lower lip. On its surface, as in the cases previously noticed, were several dried crusts. On dividing it, I found a great many enlarged papillæ, visible even to the naked eye. At Fig. 5, *a*, I have represented the appearance of one half of the tumor cut in this way. The inside of the lip was smooth in some parts, but roughened in others, by projections of these papillæ.

*Microscopical Examination.*—The exterior crusty part was formed, in this instance also, of dark epithelial cells and blood-globules. The convolutions, which gave to the tumor a mulberry appearance, consisted of epithelial cells held together by fibrous tissue. A section from the middle of the tumor, showed large epithelial and globular cells. Some of these epithelial cells were round, (Fig. 5, *b*) with large nuclei, even nucleoli, and resembled cancer cells very much, for which, if seen alone, they might readily be taken.

Fig. 5,

*a.* Interior of the tumor showing enlarged papillæ, natural size.

*b.* Round cells with nuclei and nucleoli; magnified 220 diameters.

*c.* Fibrous bands enclosing cells.

*d.* A papilla, treated with acetic acid; magnified 200 diameters.

The softer parts of the tumor were composed of globular cells and granules; the harder portions were made up of fibrous tissue and of cysts, filled with globular cells. The interior of the lip, which as I have said before, was much roughened, showed fibrous bands loosely enclosing epithelial cells. (Fig. 5, *c.*) These fibres supported the cells, and would, if condensed by pressure, have formed cysts around them. The papillæ found in the central part of the tumor were very distinct. I succeeded in placing an entire one under the field of the microscope, and was very much struck with its apparent fibrous structure; it was only after allowing acetic acid to act on it for some time, that I found it to consist (Fig. 5, *d*) of flattened epithelial cells, with a few globular cells and free nuclei.

*Measurement of Cells found in this Tumor.*

| | |
|---|---|
| Epithelial cells from the middle of tumor | Cell 1-1000th, nucleus 1-5000th part of an inch in diam. |
| Round cells with nucleus and nucleolus | Cell 1-1200th, nucleus 1-3950th, nucleolus 17900th. |
| Globular cells . . . . . . | Cell 1-4800th and 1-3750th. |
| Epithelial cells on papillæ . . . | Cell 1-1300th, n. 1-5200th. |

Case V. Mr. W. æt. 83, consulted Dr. Pancoast, Dec. 21st, 1850, for a tumor on his right cheek. Six months previously, he first noticed a little red pimple, which gave rise to intense itching. He scratched it continually; and the consequence was that it became inflamed and grew very rapidly. Dr. Pancoast removed the tumor Dec. 29th; the wound healed well, so as to leave scarcely a visible cicatrix, and the patient has had no return of the disease.

The tumor (Fig. 6 *a*) was about the size of a dime, red, vascular, and elevated above the surrounding tissue; its interior was softened and contained a juice very much like that seen in encephaloid cancer.

*Microscopical Examination.*—Under the microscope, the red vascular surface was found to consist of epithelial cells, granules, and blood globules. In a layer beneath were many globular cells, and cysts, mostly filled with granules. A deeper section

Fig. 6.

*a.* The tumor after its removal; natural size.

*b.* Cysts filled with granules; magnified 220 diameters.

*c.* Small dark epithelial cells from the softened part of the tumor.

*d.* Large globular cells.

showed distinctly nucleated epithelial cells, like those seen Fig. 4 *b*, and a great many large granules, which are always present in tumors of the face. The juice in the interior of the tumor consisted mainly of dark epithelial cells (Fig 6 *b*), and very large globular cells (*d*), measuring more than the 1-4000th part of an inch, and larger than I have yet seen them in any tumor.

Case VI. Miss H. G——, æt. 22, was operated on by Dr. Mütter, Nov. 19th, for the removal of a cupped tumor from the chin, about the size of quarter of a dollar. The tumor first attracted attention thus: About ten weeks before the operation, the patient, whilst sitting in church, was annoyed by intense itching at the incisive fossa, which was aggravated by rubbing. Upon coming home, she noticed the skin in the fossa above the chin to be dry and covered with scales like those of pityriasis. Her pride prompted her to cover the spot with court plaster, which served to increase the irritation, without in any degree alleviating the itching. After a few days the skin became broken, and the itching was supplanted by a deep smarting pain. Irritating ointments and washes were unremittingly applied, and changed by the successive advice of a number of physicians, quacks, and old women, until the itching spot became an open sore, with hard elevated edges, enclosing an area of flabby granulations, from which a thin fluid was discharged, occasionally intermingled with pus. It is well to remark that the patient has always been very scrofulous, had then enlargement of the glands under the chin, and a spot in the axilla resembling the one described in its first stage; it had, however, not been subjected to irritating ointments and washes. The operation consisted in taking out the whole of the tumor from the chin by a circular incision, and then bringing the sides of the wound together by means of harelip pins: the incision healed well and almost throughout by the first intention.

Dec. 16th, Dr. Mütter took out of her lip again a little hard tumor, which had formed since the first operation. Since this period no return has taken place; the enlarged glands under the chin and in the axilla, have, as far as I could learn, disappeared.

The first tumor was, as before observed, surrounded with a hard distinct ring; the interior was red and granulated; the tissue around its base was red, sensitive, and presented numerous minute vessels ramifying beneath the cuticle. The second tumor was hard and about the size of a pea.

*Microscopical Examination.*—A piece from the exterior of one of the granulations of the tumor on the chin, contained epithelial cells with large double nuclei (Fig 7 *a*), some with nucleoli. These nuclei measured the 1-3500th of an inch in diameter, and were very distinct. Besides these cells I found great masses of globular cells (*b*), which were dark and indistinctly nucleated. The hard ring around the tumor consisted of fibrous tissue, with free nuclei and globular cells infiltrated into it. (Fig. 7, *c.*) In the tissue next to the hard ring, I found pale, non-granulated, fibro-plastic cells (*d*) and globules, also (*e*) a small fibro-plastic mother-cell. These fibro-plastic cells resembled cancer cells very much; they were, however, more indistinct, and had fainter outlines than cancer cells usually have. The tumor taken from

Fig. 7.

*a.* Large cell with double nucleus, magnified 220 diameters.

*b.* Dark globular cells.

*c.* Fibrous tissue with cells infiltrated into it; from the ring surrounding the tumor.

*d.* Fibro-plastic cells.

*e.* A fibro-plastic mother-cell.

the lip was composed of small but distinct epithelial cells, of globular cells, and of pale cells, which I took to be fibro-plastic.

This tumor was very interesting in many respects. Firstly, from the rapidity of its growth; secondly, from being accompanied with enlargement of the glands; thirdly, from its apparent return; and fourthly, from its partially obscure microscopical character, some of the elements of which (Fig. 7, *e*) might with a little more distinctness, have caused it to be regarded as a cancerous tumor. It was, however, not cancerous. The rapidity of its growth was caused by the irritants applied. Its return was purely local; very probably a few cells had been infiltrated into the tissue of the lip, before the tumor of the chin was removed. The enlargement of the glands was not owing to the presence of the tumor, but more to the scrofulous constitution of the patient; the cells, too, were in their character more like those appertaining to a benign tumor.

CASE VI. H. P——, æt. 73, a shoemaker by trade, came, Oct. 12th, 1850, to the Clinic of the Jefferson Medical College, with an apparently carcinomatous tumor near the region of the umbilicus. He had, to use his own words, never known a day's sickness. Seven or eight years before, he noticed a small tumor forming on the part against which he had been in the habit of wearing his board, whilst working. It gave him no pain but produced a continued itching, which he relieved by scratching. A year before its removal it began to ulcerate, and was very troublesome to him, especially whilst working. It was taken out, Oct. 13th, by Dr. Pancoast. Up to Dec. 12th, 1851, the patient remained well; the disease had not returned.

*Microscopical Examination.*—The external surface of the tumor was composed of small dark epithelial cells. Beneath this were large masses of straight white fibrous tissue, with transverse fibres crossing it; a great many pale fibro-plastic cells, like those Fig. 7, *d*; and in some parts epithelial cells and globular cells. The epithelial cells formed in the centre of the tumor were like those on the surface (Fig. 6, *c*), dark and with small nuclei; many of them were not nucleated at all. The tumor was a mixture of a fibro-plastic and epithelial tumor, and was interesting from the fact, that it was evidently caused by direct pressure.

CASE VIII. Mr. E——, æt. 62, a robust healthy man, noticed, 23 years ago, a little white spot in the middle of the tongue, which produced continually a sense of roughness, and gave rise to great inconvenience in swallowing, but not to any constant pain. This spot gradually enlarged and several others formed in neighboring portions of the tongue. He applied for advice to a great many physicians, who cauterized the part, but did not prevent the disease from extending. A year ago he became the patient of Dr. Mütter, who cut out the little white lumps several times. This treatment proved very successful: the small white spots have totally disappeared, and he swallows with ease, which formerly he could only effect with great difficulty.

The part, when removed, consisted of a whitish, creamlike looking substance, sticky to the touch and of a crumbling nature. The top had a crusty appearance, darker than the centre, and elevated above the surrounding substance of the tongue.

*Microscopical Examination.*—I have at different times examined the whitish substance removed, and always found the same elements. The top consisted of dried-up epithelial cells, (Fig. 3, *a*); beneath that, epithelial cells with large nuclei, (Fig. 3, *b*); granules and globular cells. On one occasion I observed cells infiltrated into the muscular fibre of the tongue; and this is the probable cause of the apparent return of the disease after the removal of some of the parts; which return, however, was always entirely local.

As a well-marked case of a cancerous tumor, I have selected the following:

Case IX. T. V——, æt. 53, a pale, sickly-looking man, was operated on at the Clinic of the Jefferson Medical College, on the 1st of February, 1851, for a cancer of the lip, which had been coming for four months, and was attended with sharp lancinating pains. A lymphatic gland of the lower border of the jaw was found slightly enlarged, but the patient refused to have this taken out. On the 19th of March, as he thought the gland was growing very rapidly, he appeared willing for a second operation. At this time there was a tumor under the chin, formed by several enlarged glands, and connected with the submaxillary, which itself was hard and about the size of a walnut. A semilunar incision was made by Dr. Pancoast, over the tumor, and the parts opened. The disease proved as extensive as was anticipated. The sublingual and adjoining lymphatic glands, as well as the sub-maxillary, were found involved, and were accordingly removed. The incision healed well, but seven weeks after the cicatrix opened, and a reddish fungous mass came from the seat of the wound. This mass grew rapidly, and attained the size of a fist; the discharge that came from it was very offensive. A lump also formed behind his ear and one on his neck; both grew quickly, were attended with lancinating pain, and opened three weeks after their first appearance. The patient was very much emaciated, but bore his sufferings with fortitude. A week before his death, he undertook a journey to a quack in New Jersey, who applied very energetic caustics to the parts affected, and caused them to inflame violently; a circumstance which no doubt hastened the fatal termination. He died Dec. 21st, 1851; the tumor on the neck was found to have extended below the clavicle.

The tumor on the lip was not very large; it had begun to ulcerate on the surface; the submaxillary gland taken out in the second operation, was hardened, but presented no other morbid appearance to the naked eye.

*Microscopical Examination.*—The exterior of the tumor of the lip was composed, as in epithelial tumors of the lip, of epithelial cells; in the part that had begun to ulcerate, there were pus cells (Fig. 8, *a* and *b*), and a few small cancer cells. The central part consisted of granules, compound granular cells, and of many cancer cells with large nuclei. The internal surface of the tu-

Fig. 8.

*a.* Pus cells magnified 220 diameters.

*b.* Pus cells treated with acetic acid.

*c.* Cancer cells from the interior of the tumor.

*d.* Cancer cells in loose cysts, from the submaxillary glands.

mor contained fibrous tissue and cysts, filled with cancerous matter. In some sections I found the muscular fibre, entering into the composition of the lip, infiltrated with cancer cells, but yet preserving its natural appearance; in others it was completely altered in structure,—a change which I have frequently noticed in cancerous tumors. A section from the submaxillary gland proved this, too, to be cancerous. It contained many cancer cells and granules, like those in the tumor of the lip. In some parts were strong fibrous tissue; in others, again, a very delicate fibrous tissue forming loose cysts filled with cancerous matter. (Fig. 8, *d*). The small glands, too, connected with the submaxillary, were cavernous and showed under the microscope the same elements.

## Figures and Tables

**Fig. 3 f1:**
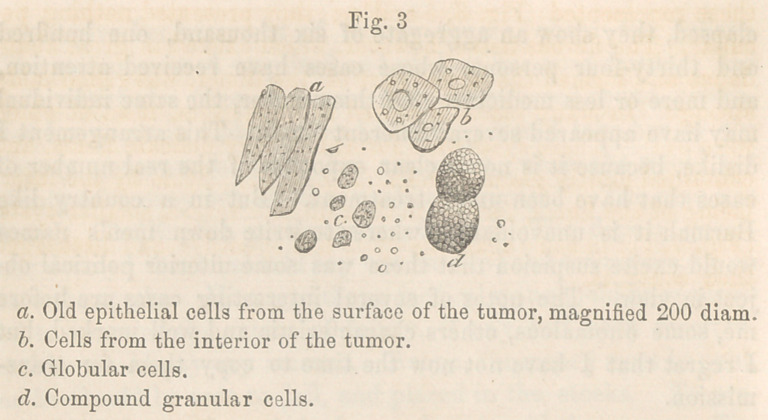


**Fig. 4. f2:**
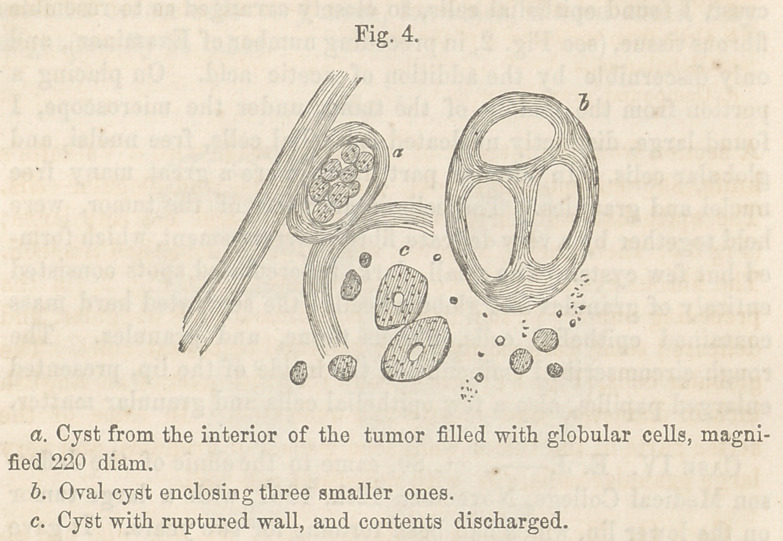


**Fig. 5, f3:**
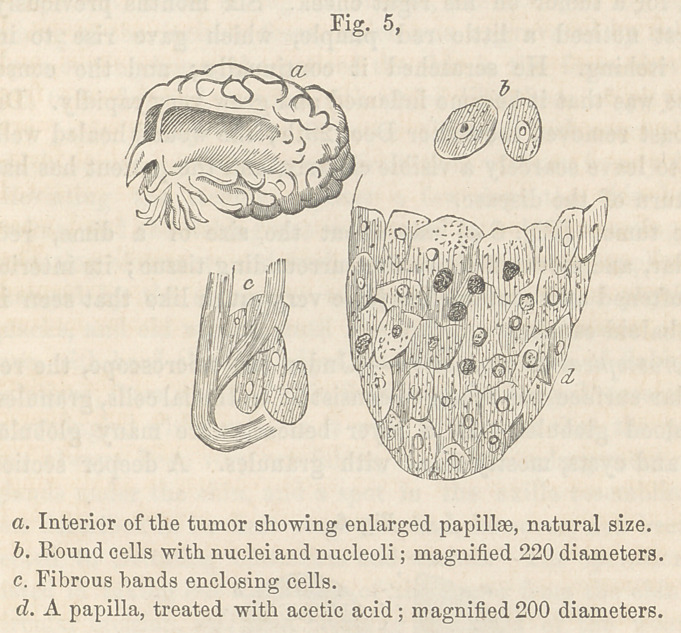


**Fig. 6. f4:**
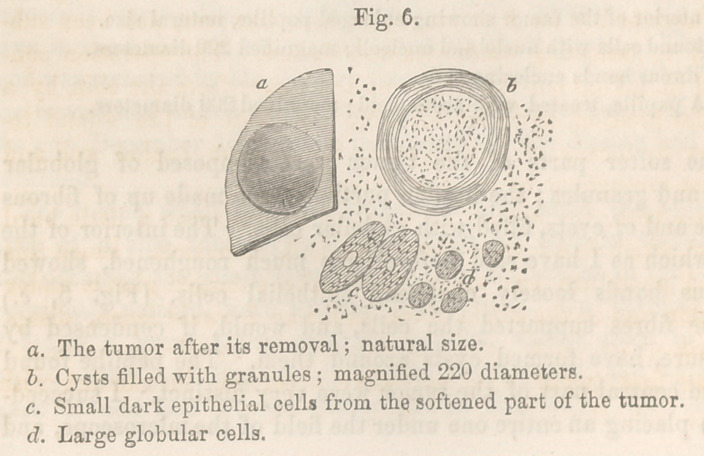


**Fig. 7. f5:**
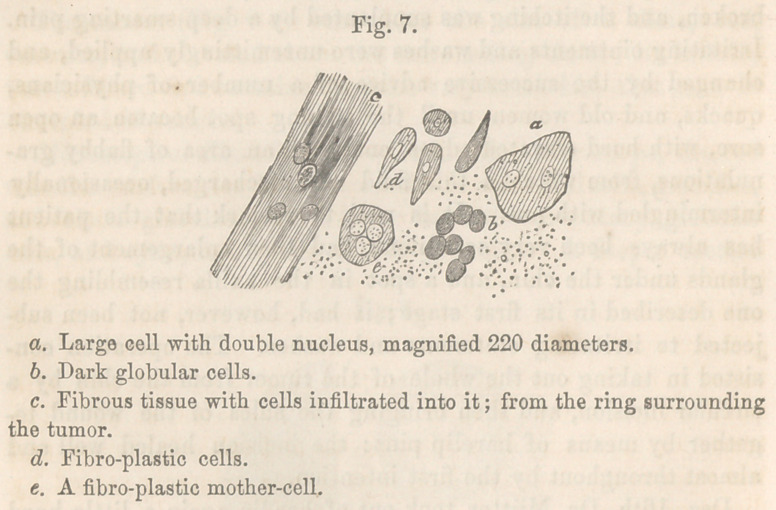


**Fig. 8. f6:**